# Advancing scalability and impacts of a teacher training program for promoting child mental health in Ugandan primary schools: protocol for a hybrid-type II effectiveness-implementation cluster randomized trial

**DOI:** 10.1186/s13033-022-00538-7

**Published:** 2022-06-20

**Authors:** Keng-Yen Huang, Janet Nakigudde, Elizabeth Nsamba Kisakye, Hafsa Sentongo, Tracy A. Dennis-Tiwary, Yesim Tozan, Hyung Park, Laurie Miller Brotman

**Affiliations:** 1 Department of Population Health, New York University Grossman School of Medicine, 227 East, 30th St, 7th Floor, New York, NY 10016 USA; 2 College of Health Science, Makerere University, PO Box 7072, Kampala, Uganda; 3 Uganda Ministry of Education and Sports, Embassy House, PO Box 7063, Kampala, Uganda; 4 Uganda Ministry of Health, Plot 6 Lourdel Road, PO Box 7272, Kampala, Uganda; 5 Department of Psychology, Hunter College of the City University of New York, 695 Park Avenue, New York, USA; 6 College of Global Public Health, New York University, 708 Broadway, New York, USA

**Keywords:** School Program, Mental Health, Prevention, Effectiveness-implementation, Uganda, Sub-Saharan-Africa, Scale-up strategy, Task shifting, Hybrid-type II, LMIC

## Abstract

**Background:**

Children in low-and-middle-income countries (LMICs) are facing tremendous mental health challenges. Numerous evidence-based interventions (EBIs) have been adapted to LMICs and shown effectiveness in addressing the needs, but most EBIs have not been adopted widely using scalable and sustainable implementation models that leverage and strengthen existing structures. There is a need to apply implementation science methodology to study strategies to effectively scale-up EBIs and sustain the practices in LMICs. Through a cross-sector collaboration, we are carrying out a second-generation investigation of implementation and effectiveness of a school-based mental health EBI, *ParentCorps Professional Development (PD),* to scale-up and sustain the EBI in Uganda to promote early childhood students’ mental health. Our previous studies in Uganda supported that culturally adapted *PD* resulted in short-term benefits for classrooms, children, and families. However, our previous implementation of *PD* was relied on mental health professionals (MHPs) to provide *PD* to teachers. Because of the shortage of MHPs in Uganda, a new scalable implementation model is needed to provide *PD* at scale.

**Objectives:**

This study tests a new scalable and sustainable *PD* implementation model and simultaneously studies the effectiveness. This paper describes use of collaboration, task-shifting, and Train-the-Trainer strategies for scaling-up *PD,* and protocol for studying the effectiveness-implementation of *ParentCorps-PD* for teachers in urban and rural Ugandan schools. We will examine whether the new scale-up implementation approach will yield anticipated impacts and investigate the underlying effectiveness-implementation mechanisms that contribute to success. In addition, considering the effects of *PD* on teachers and students will influence by teacher wellness. This study also examines the added value (i.e. impact and costs) of a brief wellness intervention for teachers and students.

**Methods:**

Using a hybrid-type II effectiveness-implementation cluster randomized controlled trial (cRCT), we will randomize 36 schools (18 urban and 18 rural) with 540 teachers and nearly 2000 families to one of three conditions: *PD* + *Teacher-Wellness (PDT), PD* alone (*PD*), and Control. Primary effectiveness outcomes are teachers’ use of mental health promoting strategies, teacher stress management, and child mental health. The implementation fidelity/quality for the scale-up model will be monitored. Mixed methods will be employed to examine underlying mechanisms of implementation and impact as well as cost-effectiveness.

**Discussion:**

This research will generate important knowledge regarding the value of an EBI in urban and rural communities in a LMIC, and efforts toward supporting teachers to prevent and manage early signs of children’s mental health issues as a potentially cost-effective strategy to promote child population mental health in low resource settings.

*Trial Registration*: This trial was registered with ClinicalTrials.gov (registration number: NCT04383327; https://clinicaltrials.gov/ct2/show/NCT04383327) on May13, 2020.

**Supplementary Information:**

The online version contains supplementary material available at 10.1186/s13033-022-00538-7.

## Contributions to the literature

- This study investigates a new scalable and sustainable *PD* implementation model (that integrates the collaboration, task-shifting, and Train-the-Trainer strategies). This study examines whether the new scale-up implementation model will yield anticipated implementation and effectiveness outcomes as the original *PD* implementation model. Knowledge gained from this scale-up research has the potential to be applied to scale-up other similar school-based EBIs in low resource settings.
- Guided by the implementation science framework and methodology, the study investigates the effectiveness and underlying effectiveness-implementation mechanisms of an EBI (*PD*) for promoting primary school students’ mental health. Knowledge gains from this theory-guided EBI implementation can inform effective approaches to implement and scale up school-based mental health EBIs in other LMICs.
- This study also investigates the role of teacher wellness and studies whether intervention on teacher wellness will improve the uptake, effectiveness, and sustainment of the EBI.

## Background

Children under age 15 in Uganda comprise 47% of the total population [1] and face enormous health and educational challenges [2]. More than one-quarter of Ugandan children have mental health problems [3], and only 53% achieve grade-level academic competency in 6th grade [4]. To address the mental health and educational burden in LMICs, providing population-level preventive services to promote child mental health has become a global priority. Despite emerging research suggesting the feasibility of adapting and transporting existing child mental health evidence-based interventions (EBIs) to Sub-Saharan Africa (SSA) [5–7], large-scale effectiveness research with diverse populations and sustainable and scalable approaches in SSA are still lacking. In addition, because of the shortage of mental health professionals (MHPs), most mental health EBIs in LMICs rely on a task-shifting approach for implementation (which involves redistributing mental health interventions and service implementation tasks from professionally trained mental health workers to those with less training and fewer qualifications in the mental health area of expertise [8, 9]). However, challenges related to task-shifting (e.g., workforce stress and job burnout) have rarely been addressed. For task-shifting to be successful, strategies to overcome challenges faced by the workforce and understanding mechanisms to support effective task-shifting are of paramount importance. We designed this research protocol to address EBI scaling-up, implementation challenges, and effectiveness research gaps in LMICs.

### Population-level approach to Child Mental Health Promotion in LMICs

Young children in LMICs spend a considerable amount of time in school; In Uganda, 95% of children are enrolled in primary schools (~ 23% enrollment in pre-primary schools) [10, 11]. Although the Ugandan child and adolescent mental health policy prioritizes the engagement of communities and child-serving institutions to contribute to mental health promotion efforts [12], school-based mental health interventions have not been widely applied in Uganda or in LMICs more generally. Population-level or universal school-based physical health programs have been found to be effective and cost-effective in addressing a wide range of individual, family, and service needs [13–15]. A school-based approach to mental health promotion can be feasible and a sustainable strategy to reach the majority of Ugandan children.

#### The EBI: ParentCorps

*ParentCorps* is a multi-component school-based preventive intervention that promotes early childhood mental health and development; it was built on an extensive body of cross-cultural parenting and child development research [16–23]. *ParentCorps* includes two components to support teachers and families to create environments that are safe, nurturing, and predictable for children: (1) *Professional Development (PD)* for teachers on family engagement and social-emotional learning (SEL); (2) *Family Program* to support children’s development of social-emotional competencies and self-regulation skills that are foundational for mental health. The *PD* and *Family Program* components encourage consistent use of a set of strategies by teachers and families. To achieve population-level reach and impact, *ParentCorps* is embedded in early childhood education programs as part of the normative school experience for all children. Two cluster randomized controlled trials (cRCTs) in the United States (US), one cRCT in Uganda, and one pre-post evaluation in Nepal have documented impact evidence. The US trials (included both *PD* and *Family Program*) found that *ParentCorps* resulted in a broad range of long-term benefits for children from low-income families, including better mental health and academic performance three years post-intervention [13, 17, 24, 25]. The Ugandan and Nepal studies (included culturally adapted *PD*) found that *PD* led to greater use of evidence-based strategies by teachers and short-term improvement in child mental health outcomes [7].

#### ParentCorps implementation model

In the US, *ParentCorps* MHPs provide *PD* to teachers and school-based MHPs. School-based MHPs implement the *Family Program*. Because most LMICs do not have school-based MHPs [26, 27], implementing *PD* and the *Family Program* with existing resources is not feasible or scalable. Given the resource limitations and calls to provide preventive intervention more broadly in LMICs, we carried out a series of investigations to test one of the two components of *ParentCorps—PD only*—in urban Uganda (RCT in 10 schools) [28] and rural Nepal (pre-post change in 30 schools) [29]. In both countries, we considered *PD* to facilitate task-shifting of mental health promotion from mental health professionals to classroom teachers [8, 9]. The *PD (training and coaching)* was intended to help teachers to create classroom environments that support SEL and mental health, to engage in mental health promoting interactions with students in the classroom and with parents during formal and informal interactions. The focus of *PD* about universal strategies for all children and coaching extended the application of these learnings to students and families with specific mental health needs.

Our previous *PD* implementation in LMICs was carried out in cross-agency collaboration with the Ministry of Education (MOE), Ministry of Health (MOH), and academic institutions using localized implementation models and culturally adapted contents [7, 30]. Using a train-the-trainer model, local MHPs were trained by *ParentCorps* MHPs to provide *PD* to teachers, reaching more than 300 teachers across Uganda and Nepal. In both countries, we found that *PD* provided by local MHPs led to greater use of evidence-based strategies by teachers and improved child mental health outcomes (see Additional file 1: Table S1 for *ParentCorps* implementation models and effectiveness evidence in the US and global context). Our previous research showed the feasibility of a train-the-trainer model to *PD* and a task-shifting approach to mental health promotion by teachers. Building on this promising evidence from two LMICs (in both urban and rural contexts), the current study extends the existing partnership with the Ugandan MOE and MOH and further applies implementation science methodology to study new localized scalable and sustainable strategies for *PD* implementation in both rural and urban settings. Applying implementation science methodology allows us to rigorously study implementation and system change strategies to address EBI implementation gaps and promote systematic uptake of *PD* into the school/education system to improve everyday real-world challenges in schools in LMICs.

#### Considerations for a scalable and sustainable model to implement PD in low resource contexts

To implement *PD* in a scalable and sustainable manner, system- and workforce-level strategies need to be considered. Because the education system in Uganda does not have a school mental health service structure, and the majority of Ugandan schools do not have mental health resources, the provision of accessible and sustainable preventive mental health interventions and services to reach a large number of schools requires systems-level intervention. From a workforce sustainability and EBI sustainment perspective, because *PD* in LMICs relies on a task-shifting approach to provide the preventive service in schools, challenges related to task-shifting (i.e., teacher workforce stress and job burnout) and additional support strategies need to be considered. This study applies a scalable and sustainable implementation model, which integrates two system-level strategies and one workforce-level strategy (in addition to the task-shifting strategy described above that has been integrated into *PD*) that have been identified as effective for providing public health interventions at scale in low-resources settings.

*A systems-level intervention strategy* [31]. The *Framework for Scaling Up* proposed by the World Health Organization [31] provides a guiding framework to strategically carry out systems changes for scaling-up EBIs. Our implementation work is guided by this framework to address existing child mental health policy implementation and research gaps in Uganda. Starting in 2007, the Ugandan government proposed a series of reforms aimed at strengthening the country's mental health sectors [12, 32]. Although several efforts have been made, no meaningful changes have occurred in child mental health [33]. To establish school-based mental health preventive intervention services, in 2013, our team initiated an effort to establish an implementation research partnership between US academic institutions and Ugandan academic, governmental, and NGO stakeholders. We carried out a series of child mental health epidemiological and intervention implementation studies to prepare for this scale-up [28, 30, 34]. We systematically assessed local school ecological systems (e.g., policy, practices) and identified scale-up strategies that are in line with current policies and locally available resources [31, 35]. We developed a sustainable scale-up strategy promoted by the MOE and MOH—train and build a workforce for *PD* implementation embedded within the MOE teacher-training structure (i.e., teacher-training colleges) to provide *PD* to teachers.

*A partnership strategy* [36]*.* Cross-disciplinary and cross-agency partnership strategies can be effective in overcoming systems barriers when existing structures do not have sufficient capacity for large-scale public health program implementation [35, 37, 38]. In Uganda, Teacher Training Colleges (TTCs) are core institutions that provide in-service training for teachers. Therefore, TTCs’ trainers/faculty they can serve as the key partner to provide the proposed *PD*. Most TTCs (96%) are owned and funded by the government, and all TTCs are monitored and supported by the MOE [39]. Although the MOE has recommended a holistic approach to improve teacher competencies, including strategies for promoting child mental health, such training is underdeveloped in the current system. The Principal Medical Officer at the MOH is in charge of mental health services and is responsible for overseeing public education and mental health programs across the country. Most formal collaborations between governmental agencies for mental health services and those responsible for primary/community health focus on adults. Because of a limited number of MHPs in the country and lack of child mental health training in TTCs, a formal collaboration among MOH, MOE, TTCs, and MHP training institutions (e.g., Department of Psychiatry in Medical Schools) and teachers to task-shift and task-share preventive care tasks has the potential to create a sustainable structure to train and support teachers to promote child mental health [40]. The proposed study formalizes the role and structure of an implementation partnership across teacher education and mental health training institutions and the governmental sectors (i.e., MOE and MOH) that has the potential to be sustainable over time.

*A teacher workforce support strategy (through consideration of teacher wellness).* Teachers in Uganda and other LMICs are vulnerable to job burnout and stress [41]. Data from our prior work in Uganda revealed that 79% of Ugandan teachers reported a significant burden of stress (e.g., 70% workload stress, 41% job effectiveness, 23% emotional distress, 9% job dissatisfaction-related stress). Many Ugandan teachers requested stress management support from coaches to supplement *PD.* Teacher stress can result in anger, frustration, depression, exhaustion, and job ineffectiveness, and can have negative consequences for schools, teachers, students, and importantly EBI effectiveness [42, 43]. Teachers suffering from stress and burnout may be less engaged in the *PD*, may have reduced motivation and ability to apply evidence-based strategies over time, or may experience difficulty in sustaining the added responsibility resulting from task-shifting. This effectiveness-implementation study tests a brief stress-management intervention (Teacher-Wellness or T-Wellness), as a complement to *PD* (described below), and examines the underlying mechanisms through which teacher stress and stress management may facilitate or hinder the effectiveness, uptake and sustainment of evidence-based practices.

### Effectiveness-implementation aims and hypotheses

This study aims to investigate the effectiveness and underlying effectiveness-implementation mechanisms of *PD* when the intervention is implemented with a new scalable and sustainable model [31, 44]. Given that a high percentage of Ugandan teachers experience occupational stress that may threaten *PD* uptake, effectiveness, and sustainment, we will also test a teacher stress management package, T-Wellness, as an enhancement to *PD*. The proposed protocol applies a Hybrid-Type II effectiveness-implementation 3-arm cluster-randomized controlled trial (cRCT) [45]. The Hybrid design enable researchers to simultaneously study the outcomes of effectiveness and implementation of P*D* + *T-Wellness (PDT) and PD alone (PD*) relative to *control*. This study will generate new knowledge to improve uptake, scale-up, and effectiveness for a new scale-up approach of EBI implementation in LMIC contexts.

Three specific aims are as follows:

1. To examine the short- and longer-term effectiveness of *PD* and *PDT* on teacher evidence-based practices and child mental health outcomes when the EBI is implemented using a scalable model. *Hypotheses*: (a) *PD* is more effective than Control; and (b) *PDT* is more effective than *PD* alone; and (c) *PDT* has a more favorable cost-effectiveness ratio than *PD* alone.
2. To examine effectiveness mechanisms and theory of change underlying *PD* and *PDT*. *Hypotheses*: The mediational mechanisms of *PD* and *PDT* will be supported. Specifically, *PD* will result in greater use of evidence-based practices by teachers and this will in turn result in improved child outcomes. *PDT* will increase teacher stress management and evidence-based practices and together these will result in improved child outcomes.
3. To examine the implementation contextual factors and moderation mechanisms that contribute to teachers’ uptake and sustainment of evidence-based practices within *PD* and *PDT* schools. We will assess the impact of implementation contextual factors (e.g., fidelity, teamwork alliance, leadership support climate) on teachers’ *PD/PDT* implementation outcomes. *Hypotheses*: Better fidelity, teamwork alliance, and more supportive contexts will be associated with better teacher uptake and sustainment of EBI practices.

### Theory of change

**Fig. 1:**
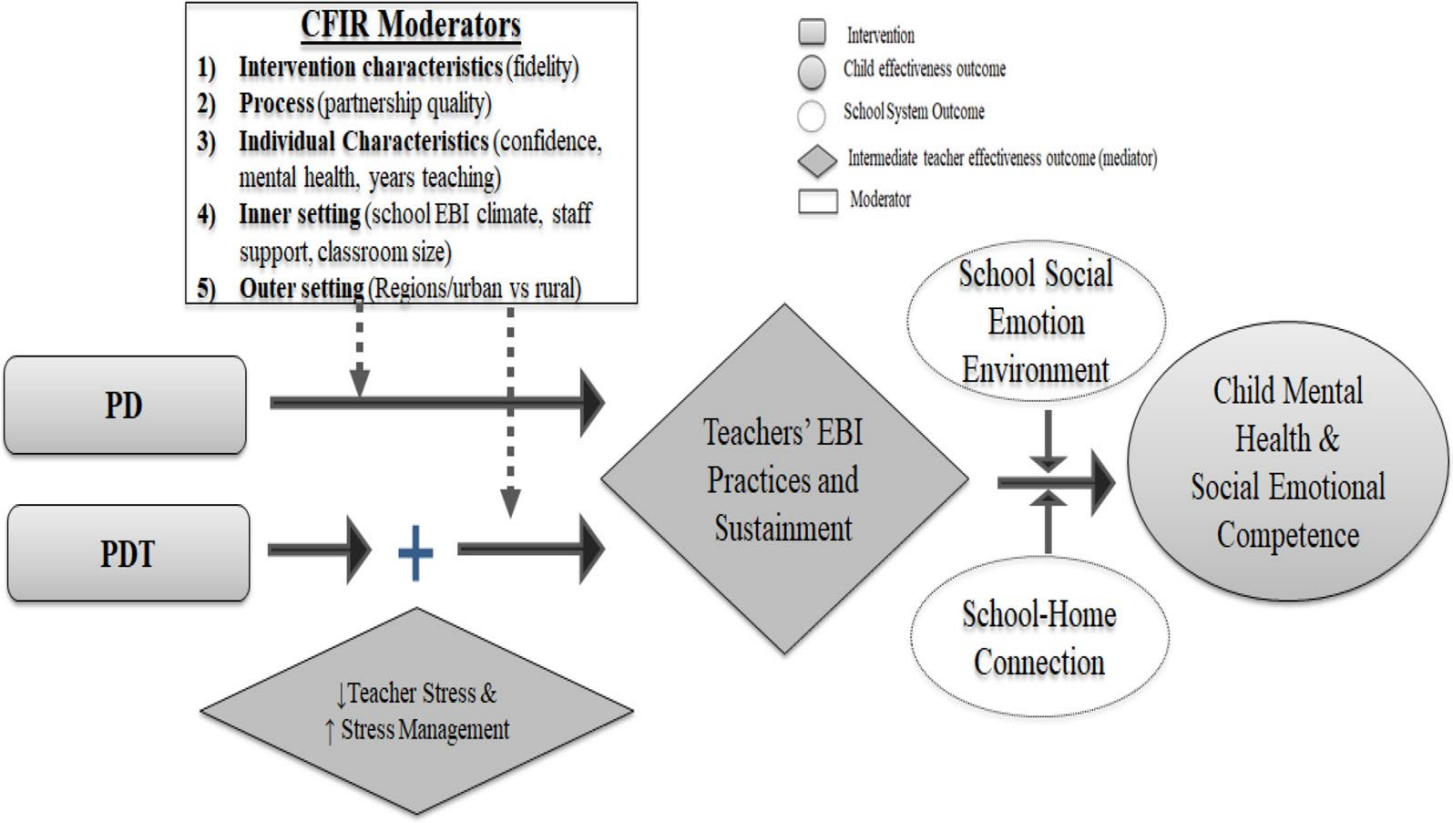
Hypothesized mechanisms for PD and PDT

The Theory of Change is shown in Fig. 1. The theory posits that *PD* will promote teachers’ use of evidence-based practices, and the impact of *PD* on distal child mental health outcomes will be mediated primarily through these practices. Similarly, we expect that *PDT* will impact distal child outcomes through both teachers’ stress management and evidence-based practices. We anticipate that *PD* or *PDT* will have immediate impact on child social and emotional skills and longer-term impact on mental health. Because *PD/PDT* will be implemented in diverse urban and rural contexts, our theory of change also considers potential moderators from the Consolidated Framework for Implementation Research (CFIR) [46]—*individual, intervention implementation, and school internal and external contexts*.

## Methods

### Trial design

We employ a matched-pair cRCT design and mixed methods evaluation data collection. For reporting, we follow the Consolidated Standards of Reporting Trials (CONSORT) for cRCT designs [47] and the Standard Protocol Items: Recommendations for Interventional Trials (SPIRIT) guidelines [48]. We will conduct a Hybrid Type II effectiveness-implementation trial [45] in two regions in Uganda. The school will be the unit of randomization because the program is applied at the school level and builds a “school community” of teachers to promote student mental health. The three-arm cRCT design allows us to simultaneously test *PD* effectiveness and study the added value of the T-Wellness to address teacher stress, which is a critical challenge to practical task-shifting effectiveness and sustainment. In addition, the Hybrid design, which considers CFIR domains of implementation contexts (listed in Fig. 1), allows us to rigorously study other EBI effectiveness-implementation mechanisms, which can further inform decisions about optimal deployment and the generalizability of impact, and may accelerate the introduction of other valuable innovations into practice [45]. To have a more comprehensive understanding of possible underlying implementation and effectiveness mechanisms, we also plan to conduct *qualitative interviews and focus groups*, which will purposefully select *PD/PDT* trainers, teachers, and parents. The study has been approved by the Institutional Review Boards of New York University Grossman School of Medicine (i20-00117), Makerere University (REC REF 2020-143), and Uganda National Science and Technology (HS1057ES).

### Participants

#### School recruitment and randomization

**Fig. 2:**
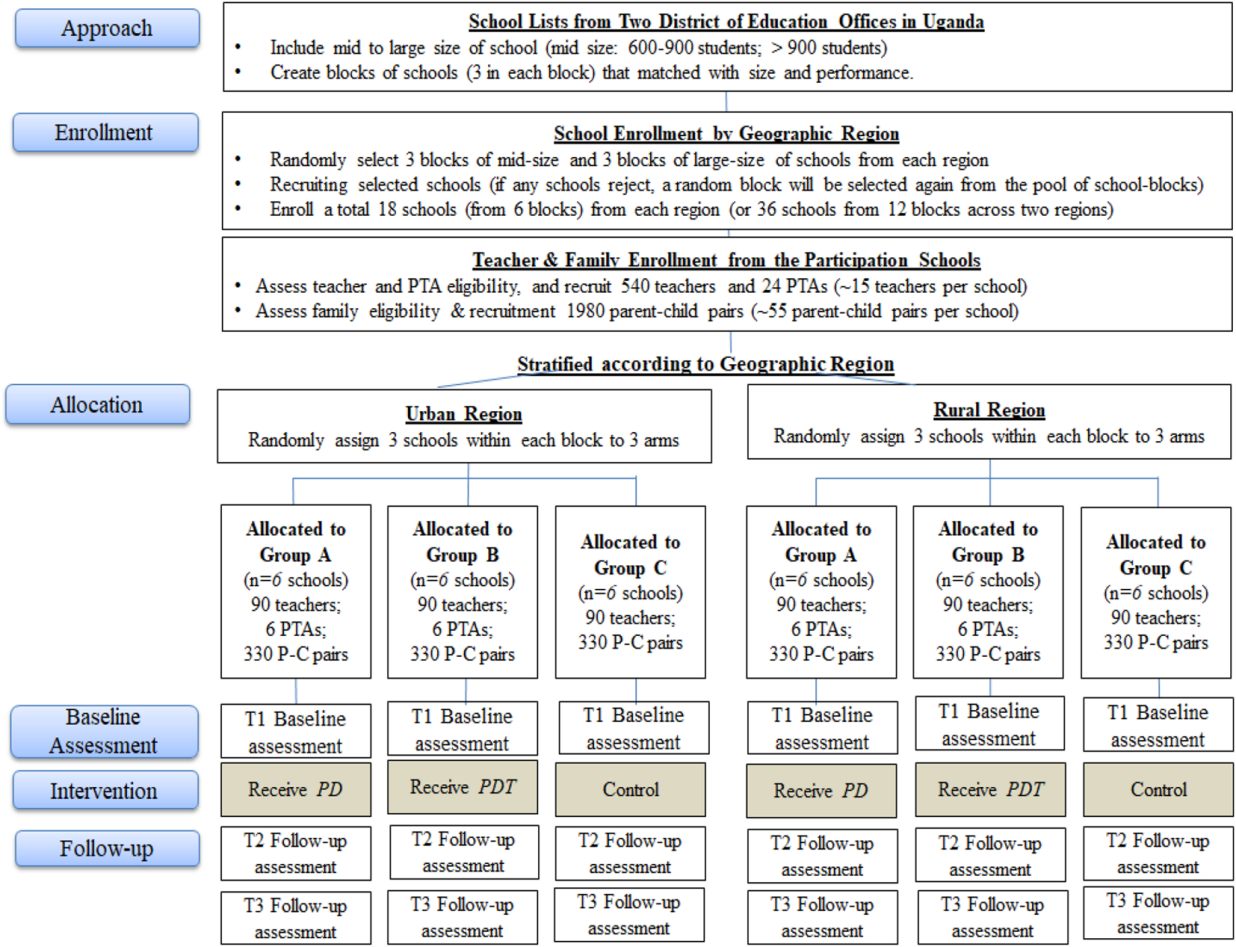
Diagram of enrollment, randomization, and follow-up

Primary schools in targeted Kibuli (urban) and Hoima (rural) districts will be identified from governmental school lists. These districts were selected based on MOE leaders’ assessment of high need. To ensure approximately similar school characteristics in three conditions across two geographic regions, a *stratified-block randomization procedure* will be applied (Fig. 2) [49]. A statistician who is unfamiliar with study schools will first match the schools on school size (teacher/student numbers) and school quality/performance (based on MOE data) within regions to ensure similar characteristics in urban and rural regions. Eighteen schools in 6 matched blocks (with blocks of size 3) from each region will be selected. Principals will be invited to attend information sessions hosted by the Ugandan study team. During recruitment sessions, principals will be provided with details of study requirements and intervention implementation procedures. They will have an opportunity to ask questions and also complete a questionnaire on school demographics, commitment, willingness to facilitate data collection, and ability to allocate staff time to participate in the study. School principals who express interest and agree to allow teachers’ voluntary participation will be eligible and will be consented. A total of 36 schools will be included in the effectiveness study. Computer-generated random numbers will be used to decide the randomization allocation sequence. Within each block (of size 3), one school will be randomly assigned to *PDT*, one to *PD*, and one to Control (receiving child mental health materials approved by MOH/MOE). The intervention and evaluation activities will be carried out in two consecutive cohorts using the two-cohort approach because it allows for building the capacity of local TTC trainers to carry out *PD* in a real-world context and provides time for TTC trainers to develop practice competency. Cohort 1 schools (n = 18; 9 urban and 9 rural) will start in 2021, and Cohort 2 schools (n = 18) in 2022. Schools from both cohorts will be actively involved for two years.

#### Teacher recruitment

All pre-primary and primary grade 1–4 teachers and teaching assistants, serving students between the ages of 3 and 10 years, will be eligible to participate. We include multiple grades because teachers in Uganda teach a wider age range of classes. Teachers’ participation will be completely voluntary, with no consequence for opting out. Based on the 100% enrollment record from our prior cRCT, we anticipate that nearly all eligible teachers will sign up for the study. We anticipate that 540 teachers from 36 schools will participate in the evaluation; and 360 intervention school teachers will receive *PD* (180 with and 180 without T-Wellness). Based on enrollment from our prior study in Uganda, we anticipate that nearly all teachers will sign up for *PD* (i.e., > 90% will participate in *PD*). Based on feedback from stakeholders, we will also recruit two parents from the Parent-Teacher-Associations (PTAs) in intervention schools to be part of the school-based team to support teachers to facilitate parent involvement (e.g., through sharing evidence-based knowledge and parenting strategies with families during parent-teacher conferences).

#### Parents and child recruitment

Students attending pre-primary and primary grades 1–4 (ages 3–10 years) and their parents/primary caregivers will be eligible to participate in the study. Given the large numbers of students in schools, research staff will randomly select 10% of students and families from each school (based on student lists provided by schools) and complete assessments over two years [50, 51]. A total of 1,980 families from 36 schools (averaging 55 families/per school) will participate in the study. Teachers will be informed of the students randomly selected for participation in the assessments and asked to introduce the study staff to the selected families. Primary caregivers from the selected families will be invited to consent for interviews, and for research staff to carry out assessments with their child. Children with parental consent will be asked to assent to the study. Although we only evaluate a subset of the sample, an estimated 13,200 students across 24 intervention schools will be exposed to *PD*. Teachers and parents who participate in the study, will receive a small incentive for their time.

#### Local PD implementers/trainers and MHP supervisors

A total of 8 Ugandan TTC trainers (4 from each TTC) and 4 MHP supervisors will be recruited and trained to implement and support *PD*. TTC trainers will be required to have a minimum of university level of education and two or more years of experience in teacher training. MHP supervisors will be clinical psychologists or mental health counselors with at least master’s degrees and two years of experience or psychiatric nurses (with at least a bachelor’s degree and 5 years of clinical experience). They will be recruited from local universities or mental health facilities. TTC trainers and MHPs who agree to participate will be asked to provide written informed consent, which will allow the research team to gather fidelity and competency data (self-reported, audio, or observational data) with their assistance.

#### Subsample for the qualitative study

Subsamples of study participants from the intervention schools will be selected to participate in qualitative interviews or focus groups aimed at better understanding the underlying mechanisms for the effective implementation and sustainment. For each study cohort we will carry out interviews and focus groups with *PD/PDT* trainers (n = 8), teachers and parents (n = 40; 20 from *PD* and 20 from *PDT* schools across urban and rural sites). Qualitative data will be conducted twice (post *PD/PDT* intervention and a year after the intervention).

### Sample size and power

We conducted power analyses for child and teacher effectiveness outcomes, assuming an intention-to-treat (ITT) analysis. The power calculation is estimated primarily based on: (i) the statistical analysis approach planned for this study (linear mixed effect models [52]); (ii) the expected magnitude of the effects for the *primary child and teacher outcomes* from our prior Ugandan *PD* study (i.e., child mental health *d* = 0.39; child social emotion competency *d* = 1.08; teacher practice outcomes-observed *d* = 0.55 and self-report *d* = 0.32); and (iii) detectable effects with 80% power of two-sided significance tests with α = 0.05. In cRCT designs, the detectable effect sizes depend on the usual study design parameters, as well as the cluster size, N, and the cluster effect, i.e., the intra-cluster correlation coefficient (ICC). Detectable effects also depend on the test used (e.g., a test that accounts for baseline outcome or examines effectiveness with repeated observations is more powerful) [52]. We anticipate that 540 teachers (or 180 per intervention condition) and 1980 parent–child pairs (or 660 per intervention condition) from 36 schools will participate in the study. We estimate power (for detectable effect size) based on the total sample (1320 families and 360 teachers for two comparison conditions [1 intervention and 1 control]), as well as based on the sample from one region (660 families and 180 teachers for two conditions) with one or two post-intervention outcome evaluations, and assuming 20% loss of sample by Time 3. Additional file 1: Table S2 gives the detectable effects for a range of cluster/ICC values and multiple scenarios. In the most conservative scenarios when the ICC = 0.05, for 24 school clusters (2-condition comparisons across two regions), the detectable effects are *d* = 0.14 − 0.22 for child outcomes, *d* = 0.18 − 0.38 for teacher intermediate outcomes; and for 12 school clusters (2-condition comparisons in one region), the detectable effects are *d* = 0.19 − 0.42 for child outcomes, *d* = 0.25 − 0.55 for teacher intermediate outcomes. This study will have sufficient power to detect impacts that are meaningful and realistically achievable.

### Description of intervention and scalable implementation approach

#### The PD implementation approach

**Fig. 3:**
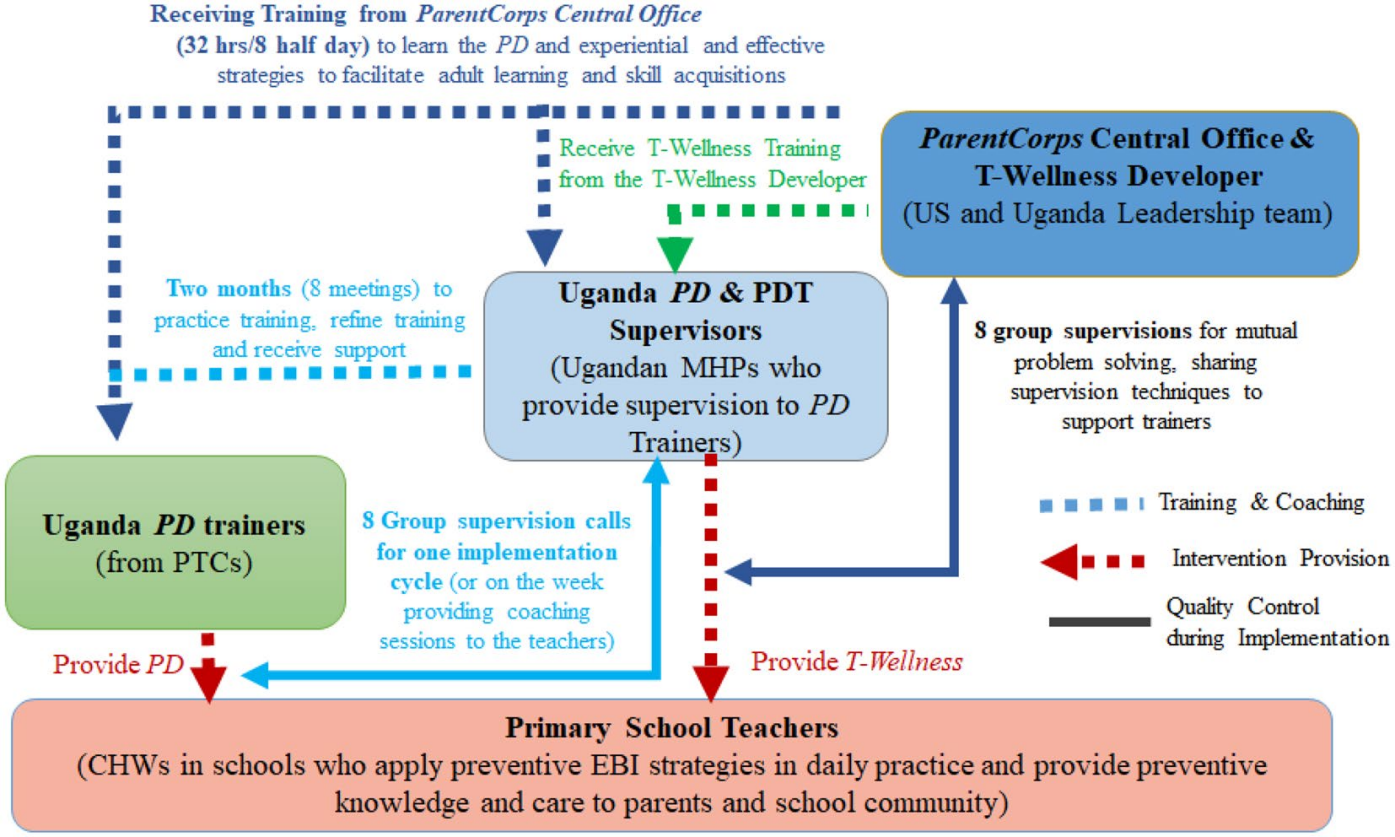
Train-the-trainers and supervision model for scalable EBI implementation

The scale-up approach to *ParentCorps PD* implementation relies on Train-the-Trainer and a dynamic multi-layered supervision model [53]. As shown in Fig. 3, an experienced clinical team from the *ParentCorps* Central Office in the US provides comprehensive virtual training and ongoing supervision to four Ugandan MHPs, including two who were previously trained and participated in the previous *ParentCorps PD-Ugandan* study. The four-person Ugandan clinical team will oversee local implementation efforts including supporting and supervising the TTC Ugandan *PD* trainers/facilitators. The *ParentCorps* clinical team from the Central Office in the US will provide virtual training to 8 TTC Ugandan *PD* facilitators (32 h over 8 half days) and the Ugandan clinical team will provide live ongoing supervision. Over a 2-month period (~ 8 weekly meetings) working with the Ugandan clinical team, the TTC Ugandan *PD* facilitators will practice, receive feedback and refine aspects of *PD* for the local context prior to the first round of implementation. Finally, with ongoing supervision from the Ugandan clinical teams (8 live group supervision sessions), the Ugandan TTC facilitators will provide *ParentCorps PD* (21 h over 3 days) to the Ugandan primary school teachers and 8 one-h of coaching sessions over a 2–3 month period.

#### Teacher-wellness implementation approach

T-Wellness was co-developed by US and Ugandan investigators. We adapted evidence-supported strategies for teacher stress and burnout management [54, 55] based on our review of over 60 teacher wellness and social-emotional interventions published in the past 10 years. We developed a brief T-Wellness intervention that includes common stress management and workplace burnout stress management/cognitive-behavioral skills. A small pilot with 50 teachers yielded anticipated effects on teacher stress and management outcomes, which and this brief intervention will be applied in this trial. *T-Wellness* is a one-day workshop and three group-support sessions (45–60 min each) for teachers. MHPs from the Ugandan clinical team will be trained by study investigators to facilitate the workshop and the support sessions to teachers. Teachers from schools assigned to the *PDT* condition will receive the 1-day workshop right after receiving *PD*. The three group-support sessions will be integrated into the *PD* coaching sessions. For implementation quality assurance, the Ugandan clinical team will receive supervision from the study investigators after each group-support session they provide to teachers.

#### Intervention conditions

##### PD

The 3 days of *ParentCorps PD* aims to help teachers to foster child social-emotional learning, strong family-school relationships and safe, nurturing, and predictable classroom environments. There are four elements that the *ParentCorps* theory of action specifies as essential processes through which *PD* strengthens teachers’ use of evidence-based practices: building authentic relationships, honoring culture, translating the science of early childhood development, and practicing self-reflection. These essential elements are measurable aspects of the quality of facilitation that complement measures of fidelity to the manuals in explaining the extent to which the program targets change. Specifically, high-quality, high-fidelity facilitation is hypothesized to support teachers in developing increased capacity as defined by (1) knowledge of evidence-based strategies; (2) awareness of self and child; (3) intentional and responsive interactions; and (4) problem-solving and support-seeking as needed (see Additional file 1: Table S1 for additional contents information).

##### PDT

*PDT* includes *PD* and 1-day teacher-wellness workshop and 3 group support sessions aim to increase teachers’ self-awareness of their stress and regulation/coping styles, and support teachers to manage stress through practicing evidence-based strategies. Prior to the workshop, teachers will be asked to complete a stress and wellness self-assessment survey using a digital toolkit. A tailored report is generated right after the assessment to share with teachers to promote self-awareness and motivation for change. During the one-day workshop, four key topic areas will be covered: (i) understanding stress and job burden; (ii) self-appraisal and identification of areas for improvement; (iii) cognitive and behavioral strategies; and (iv) teacher-to-teacher support and other additional resources. The group support sessions are to help teachers apply strategies to work toward their wellness goals.

#### Control group

Teachers in control schools will receive mental health knowledge and promotion materials. In the second year of participation (after completion of the effectiveness evaluation), control schools will *receive a one-day T-Wellness workshop* (without *PD*) and 3 follow-up group-support sessions.

### Study measures

The evaluation design is guided by the implementation outcome framework [56]. The *quantitative evaluation measures* for teacher and child effectiveness outcomes will be assessed using multiple sources (data collected from objective classroom observation, parents, teachers, and children) and across 3 time points (T1 baseline, before *PD;* T2 immediately after the PD/PTD, about 3–4 months after T1; and T3, 9–12 months after T2). The research staff responsible for *family and observation data* collection will be masked to intervention conditions. To ensure masking, we will have an independent assessment team (led by a separate research coordinator), and members will not participate in any intervention activities. We will also train the implementation team on the protocol to prevent unblinding. Table 1 lists the measures included in the study. Most of the measures have been used and validated in our previous pilot trial [28].

**Table 1:** Key study measures for effectiveness-implementation study: constructs and measures

Dimensions (assessment time)	Constructs	Measures and sources of data
Child effectiveness outcomes (T1, T2, T3)	Mental Health (Primary): Exteranalizing and Internalizing	Strengths and Difficulties Questionnaire (α = .63–.80)[57] (P); PROMIS Anger (α = .78), Anxiety/Fear (α = .90), Depression (α = .87) [58–60](P)
	Social Emotional Competency (Secondary): Emotion regulation; Relationship; Executieve functioning	Emotion-regulation (α = .90) [28] (P); & Peer relationship α = .75) [28] (P); Comprehensive Computerized Battery for Child Psychological Assessment (for executive functioning) [75] (C)
Teacher effectiveness outcomes (T1, T2, T3)	Teacher EBI Practice (Primary): PD strategies use	Teacher EBI Practices Classroom Observation (α = .68–.72) [28] (O); EBI Strategies Practice Questionnaire (α = .69–.80) [28] (T)
	Teacher Stress and Management (Primary)	Stress Questionnaire [61] (T); Responses to Stress Questionnaire [62, 63] (T); Social Support (α = .97) [64] (T). Difficulties in Emotion Regulation Scale (DERS-18) [65] (T)
	Social Engagement (Secondary): Parent and student engagement	Teacher-student relationship (C)(α = .78) [29]; Family and Teacher Relationship Quality [76] (P)
Implementation outcomes (during training and coaching sessions)	Fidelity: (1) Adherence; (2) Quality of implementation; (3) Engagement (pre- and post-training knowledge gain) (4) Exposure	(1) PD/PDT Fidelity Checklists (F) [28]: after training & coaching session; (2) Teacher Training Experience Rating: facilitator competency (knowledge, preparation, ability to control discussion, enjoyable) (rating after training and coaching)(α = .72) [28] (T); (3) EBI Strategy Knowledge (test–retest r = .35–.43) [28] (T);(4) Attendance tracking [28] (F)
	Appropriateness and Usefulness of PD/PDT	Implementation outcome measure [66] (T)
	PD/PDT implementation costs	Implementation costs for PD/PDT: actual program costs (with and without monitoring cost) [77]
Contextual moderators- in CFIR domains (T1/beginning of the PD/PDT)	Inner setting: (1) School structural characteristics; (2) School climate (culture, learning climate, leadership engagement, team work alliance)	(1) School structural characteristics (classroom size); (2) School Environment and Climate Survey (adapted from Inner-setting scale [78] and Organization Climate Questionnaire (α = .65–.85) [79] (T)
	Individual Teacher characteristics: Role clarity; Self-efficacy; Mental health	Determinants of Implementation Behavior Questionnaire (DIBQ) [80]; PHQ-4 (mental health) [81, 82] (T)
	Intervention characteristics: Acceptability, Appropriateness,	See implementation outcome measures above
	Processes: Partnership quality, Fidelity, Intervention cohort (1st or 2nd cohort)	Partnership Quality Scale (Coach-teacher relationships, support from coaches, support from teachers) [36] (T)
	Outer setting: Urban/rural region	Geographic region (urban/rural)

For child effectiveness outcomes, primary data source is parent-report (P); and secondary data source is child-report/testing (C). For teacher effectiveness outcomes, primary data sources is classroom observation (O) and teacher report (T); and secondary data sourcs are Parent (P) and child report (C). CFIR contextual data will be gathered from from Training/coaching session tracking data from facilitator report (F) and teachers-report (T)

### Effectiveness outcome measures

#### Child effectiveness outcomes

The primary outcome is child mental health (externalizing and internalizing problems), and the secondary outcome is social-emotional competency (emotion regulation, executive functioning). Primary child mental health outcomes will be assessed using parent-rated Strength and Difficulties Questionnaire (including Conduct Problem 5 items and Emotion Symptoms 5 items) [57], and PROMIS Anger (5 items), Anxiety/Fear (8 items), and Depression measures (6 items) [58–60]. Parent and child report data will be gathered. Parents of study students will be interviewed by trained research staff (using English or Luganda, lasting 30–45 min). Participating children will be assessed by trained research staff in schools (lasting about 20–25 min).

#### Teacher effectiveness outcomes

The primary teacher outcomes are (i) EBI strategy use, which will be based on objective observation by an independent observation team (primary data source *using the Teacher EBI Practices Classroom Observation* [28]) and teacher report (secondary data source using teacher-reported *EBI Strategies Practice Questionnaire* (EBI strategies/appropriate behavioral management strategies 11 items [28]); and (ii) teacher perceived stress and stress management (teacher report) assessed using *Perceive Stress Questionnaire* (9 items) [61]), *Responses to Stress Questionnaire* (including subscales Primary Control Engagement 6 items and Secondary Control Engagement 12 items) [62, 63], *Social Support* (4 items) [64] and *Difficulties in Emotion Regulation Scale* (DERS-18) (Total emotion regulation difficulties 18 items) [65]. The secondary teacher outcome will be school-home connection, teacher-family relationship, and student–teacher relationship based on parent and student reports.

### Implementation outcome measures

*Fidelity* will be measured to assess the quality of implementation. Four fidelity measures will be considered, including *adherence* (the extent to which the TTC facilitators deliver the core intervention content and as per program guidelines), *quality of program implementation* (assessed based on teacher rating of their training experience of Coaches’ competence (knowledge, preparation, ability to control discussion, enjoyable); *engagement* (assess trainees’ level of *PD* knowledge improvement from pre- to post-training; and *exposure* (measured by trainees’ attendance in *PD* and coaching sessions) [28]. *Acceptability and Appropriateness of PD/PDT* will also be measured based on teacher-report and be assessed post-training and at T2 (after completing the full cycle of *PD/PDT*) [66].

*Cost Measures.* Costs will be measured using an activity-based micro-costing approach [67] in the intervention and control clusters (school), and in the extended implementation phase again in all clusters from 6 through 12 months (n = 36 clusters). Micro-costing entails a three-step approach where we identify, measure, and value resource use for all activities in each study arm. Resource use and cost data will be collected prospectively alongside the trial. All research costs will be excluded. Cost data collection will utilize standardized cost extraction forms and procedures that have been validated in our team’s previous work in Uganda and other LMICs [68–73]. Prior to use, these tools will be tailored and customized to the *PD/PDT* context. All costs will be adjusted for inflation, discounted to the intervention start year, and presented in US dollars.

### Contextual moderators

Constructs in CFIR domains will be measured to study the moderation effect on teacher EBI strategy use outcomes. Selection of CFIR moderators is guided by factors identified in the literature as influential factors for implementation and effectiveness outcomes [46, 74]. *Inner setting* will include school structural and climate characteristics (classroom size, learning climate, leadership engagement, teamwork alliance); *outer setting* will include region (urban/rural); *process* will include partnership quality, fidelity, and cohort (1st or 2nd implementation cohort); *intervention characteristics* will include perceived *PD/PDT* acceptability and appropriateness; and *Individual teacher characteristics* will include teacher years of experience and gender.

### Qualitative assessment

To have a more comprehensive understanding of possible mechanisms, we will conduct *qualitative interviews and focus groups.* Interview guides will comprise semi-structured questions relating to experiences with *PD/PDT* and sustainment of *PD/PDT*. Participants will be asked to provide a narrative account of partnership approaches and efforts to implement and sustain *PD/PDT*, including barriers and facilitators experienced. Qualitative assessment will be conducted twice at T2 and T3. We will also conduct qualitative assessments separately for each cohort, which allows a better understanding of cohort effects and whether quality of implementation improves over time.

### Data management

All data will be managed and stored in REDCap (Research Electronic Data Capture). REDCap is a secure web application for building and managing surveys and databases for research studies, originally developed at Vanderbilt (www.project-redcap.org) with collaboration from a consortium of worldwide institutional partners. It provides automated export procedures for seamless data downloads to common statistical packages such as Excel, SPSS, SAS, Stata, and R. Access to study data in REDCap will be restricted to the specific members of the study team with authentication. Qualtrics will also be used when collecting data in the field through the offline mobile app function. When using Qualtrics offline mobile app, no identifying information will be collected. Qualtrics mobile app uses Transport Layer Security (TLS) encryption (also known as HTTPS), and data entered into the mobile app cannot be re-accessed in the front-end. Only selected staff members have access to the data in the back-end through password protected accounts. Data will be entered using only the unique study identification number. Qualtrics data will then be transferred backed into REDCap as our database management system. All final study files for analyses will be captured and finalized, ensuring that no personal identifiable information (PII), including students’, parents’ or teachers’ names and contact information, are included. Electronic data entered that include contact identifying information (e.g., master list of consenting information, contact information/address) will be securely saved, and will not be linked to the study data. There will be additional levels of protection and access restrictions to this information.

### Data analyses and statistical methods

#### Preliminary analyses

Prior to any outcome analyses, we will generate summary statistics for all data, summarizing with means and standard deviations for continuous variables and frequencies for categorical variables. Baseline equivalence between intervention and control schools will be examined. For measures that evaluate similar constructs, composite scales will be created (to minimize the number of analyses). In addition, the distribution of study variables and missing data patterns will be inspected. For participants with partially missing data, a multiple imputation strategy using a Markov chain Monte Carlo approach will be applied. We will also sequentially impute data for each wave using the predictive mean matching method separately for intervention and control groups to account for the possibility of different missing data patterns by condition [83–85]. Ten data sets will be imputed, and SAS PROC MIANALYZE will be used to combine the results for the final inference [86].

#### Analyses for aim 1

To estimate effectiveness, we will apply intention-to-treat (ITT) analyses and first focus on between-subject comparisons of intervention *vs.* Control (comparing *PD* to control, comparing *PDT* to control, and comparing *PD* to *PDT*). We will estimate the impact of *PD* on children and teachers post-intervention (T2, 4–5 months after T1) and at one-year follow-up (T3, 12 months after T1). School and class nesting will be considered, and a multiple imputation strategy will be applied to account for missing data. Linear mixed effect models [52], using SAS PROC MIXED [86], will be applied to examine short and longer-term impacts. We will first *examine the immediate impact* by modeling post-intervention outcomes (T2) as a function of intervention, adjusting for T1 outcome measures. The model accounts for the correlation between subjects (within-school and -class) by including school- and classroom-level (when appropriate) random intercepts. Next, we will *study longer-term effectiveness outcomes* (T2 to T3) by applying growth curve models and using repeated assessments over time. In these growth models, we will add time-relevant parameters to the model above, including school-level random slopes associated with time. The post-baseline scores will be modeled as a linear function of time, intervention indicator, and intervention-by-time interactions, adjusting for T1 scores and cohort.

*Cost-effectiveness analysis* of *PD* and *PDT* implementation models will be examined using approaches that have been applied in the previous school-based and parenting-based child mental health promotion research [87–90]. The analysis will center on incremental cost-effectiveness ratios (ICERs), where the numerator represents the cost difference between the intervention arms and the Control, and the denominator represents the difference in average intervention effects. To that end, the cost-effectiveness analysis of the intervention will involve examining how much the *PD/PDT* intervention costs to achieve a unit of effect relative to the control group. The effects of the intervention will be estimated using the effect sizes *d* (standardized mean difference between intervention and control groups) using an ITT approach. For the effectiveness outcomes, we will use an effect size of 0.2–0.4 as a benchmark; this corresponds to a small to medium effect size according to Cohen [91]. We will compute the per-participant cost per 0.2–0.4 SD change for each child effectiveness outcome. Reporting of the cost-effectiveness analysis will follow the Consolidated Health Economic Evaluation Reporting Standards (CHEERS) [92].

#### Analyses for aim 2

We will examine mediation mechanisms for *PD* and *PDT* separately. The analysis will be built on the Aim 1 Linear mixed effect models. For *PD*, we will examine whether the impacts of *PD* on children’s mental health are mediated through improvement in teachers’ EBI practices (primary). For *PDT*, we will examine whether impacts of *PDT* on child mental health are mediated through improvement on teachers’ EBI practices and stress management (in cognitive and behavioral domains). The intermediate teacher outcomes will be based on T1 and T2 data, and the child outcomes will be based on T2 and T3 data (to capture changes over time).

#### Analyses for aim 3

To study effectiveness-implementation moderation mechanisms, we will test whether the impacts of the intervention on teacher effectiveness and EBI practice sustainment outcomes is moderated by CFIR contextual factors. We will apply similar approaches as in Aim 1 and add the moderator and moderator-by-intervention interaction terms in the analysis. T2 implementation and T2 and T3 teacher effectiveness outcome data from the PD/PDT intervention samples will be utilized. Any significant moderators identified will suggest important factors to be intervened on in future implementation to enhance the uptake of evidence-based strategies by teachers, or improve the effectiveness of task-shifting.

#### Qualitative data analyses

For the qualitative focus group data, we will apply qualitative analysis methods. Interview data will be transcribed and analyzed using Atlas.ti software. To better understand *partnership/scalable approaches*, coding will focus on themes related to the partnership development process, the usefulness of partnership frameworks in formalizing processes, scalable strategies, intervention implementation barriers, teacher stress, and strategies for overcoming teacher stress and other practices (considering CFIR). These analyses will help identify facilitators and barriers for partnership and implementation for carrying out the effectiveness study. For *effectiveness-implementation mechanisms,* qualitative analysis will focus on themes related to implementation barriers, facilitators, and contextual factors and processes that influence teacher intermediate and child effectiveness outcomes. Coding of qualitative data will follow a *constant comparative analysis* approach, where data are analyzed for themes that reflect project aims, which are then confirmed by further data analysis, followed by a third review of the data to identify additional themes [93–95].

## Discussion

This study is the next step to our previous pilot effectiveness-implementation research. We furthers our collaboration with the MOE, MOH, TTC, mental health training institutions, and teachers in Uganda to provide preventive intervention in schools to promot early childhood students’ mental health and social-emotional competence using a potential scalable and sustainable implementation model. Our scalable implementation model utilizes a task-shifting and task-sharing cross-sector partnership strategies. We will study the effectiveness and cost-effectiveness of this potentially scalable and sustainable EBI implementation model. In addition, we investigate strategies to address a common task-shifting challenge (workforce stress/burnout) and examine whether the incorporation of a workforce stress management strategy can improve the effectiveness and sustainment of EBI practices. The cost-effectiveness analysis will inform policy and implementation decisions, which are critical for the scale-up and sustainability of EBIs in low-resource contexts. Our study builds on the increasing body of evidence on task-shifting approaches of mental health promotion, scale-up research, and workforce-related implementation strategy testing. Our scale-up model and effectiveness-implementation study is designed in partnership with the MOH and MOE, which addresses local governmental needs and has the potential to affect policy change and inform school mental health system development. The EBI/*PD* applied in this study has been tested both in the US and in LMICs (Uganda and Nepal). This study will further address numerous implementation, scaling-up, and translational research gaps through strategies testing and effectiveness-implementation evaluation. The trial started in late 2021 school year, which is unique in that the intervention will be conducted in the context of the COVID-19 pandemic. The baseline and evaluation data may inform the impacts of the COVID-19 on school staff, parents, and students. The intervention is likely to mitigate the crisis and negative impacts that the pandemic has on school communities. Lessons learned and shared by the US and Ugandan collaborators can illuminate the processes and complexities of scaling up a population-wide approach for child mental health. Furthermore, the theory-guided implementation can inform the feasibility and the relevance of the theories to be applied in LMIC contexts. The knowledge gained from this study can be applied to guide other EBI dissemination and implementation efforts that utilize task-shifting/task-sharing/cross-sectoral collaboration strategies in LMICs.

### Trial status

At the time of manuscript submission (May 2021), the trial study has not yet started. Baseline data collection is planned to commence in June 2021, and the intervention is planned to begin in June-July 2021. There is a possibility that the trial will be further delayed because of the COVID-19 pandemic.

See Additional file 1: Protocol Supplementary File.

## Supplementary Information

**Additional file 1**: **Table S1**. ParentCorps implementation models and impact evidence. **Table S2**. Power estimation for child and teacher effectiveness outcomes.

## Data Availability

Trial data will be deposited to NIMH Data Archive NDA (website http://nda.nih.gov) as part of the funding agreement.

## Abbreviations

LMICs: Low-and-middle income countries
EBIs: Evidence-based interventions
MHPs: Mental health professionals
cRCT: Cluster randomized controlled trial
PD: ParentCorps-professional development
PDT: PD + T-wellness
MOE: Ministry of Education
MOH: Ministry of Health
TTCs: Teacher Training Colleges
CRIR: The Consolidated Framework for Implementation Research
