# Supplementary material for: Advancing scalability and impacts of a teacher training program for promoting child mental health in Ugandan primary schools: protocol for a hybrid-type II effectiveness-implementation cluster randomized trial
Source: Int J Ment Health Syst. 2022 Jun 20;16:28. doi: 10.1186/s13033-022-00538-7 (PMC9206883; doi:10.1186/s13033-022-00538-7)

**Supplemental File**

***Table S1. ParentCorps implementation models and impact evidence***

|  | **United States (US)** | **LMICs- Uganda (urban) & Nepal (rural)** |
| --- | --- | --- |
| **School-based Intervention Components** | **PD** (3 days + 1:1 individual coaching) + **Family Program** (14 sessions). | **PD** (3-4 days+ a group coaching model to reduce costs and to align with value for working in groups as the “collective culture” + culturally relevant handout to facilitate discussion around EBI-strategies during parent-teacher meetings) |
| ***ParentCorps* Contents** | - **Core EBI strategies included in PD & Family Program**: Draw on social learning theory and cognitive & behavioral principles that target adults’ knowledge, skills, and confidence in an effort to reduce or prevent child social, behavioral, and emotional problems). EBI strategies include: i) proactive behavioral management strategies; ii) social emotional promotion & regulation strategies; iii) managing misbehaviors & noncooperation; iv) school-family connection - **Other EBI strategies included in PD** : i) parent engagement strategies to strengthen school-home connection & support for students; ii) individual planning for supporting high-need students; and iii) coaching support in applying the EBI strategies. | |
| **Implementation model** | Mental health professionals (MHPs) from an academic institution and Department of Education (DOE) jointly implemented PD and Family program | **Localized Partnership Implementation Models**   - **Uganda (Urban**): MHPs and psychiatric nurses implemented *PD* - **Nepal (Rura**l): MHPs, community health workers (CHWs), and District Education Office staff implemented *PD* |
| **Targets in the studies** | Ethnically diverse Black, Latino & Asian children (aged 4- 6) from low-income urban communities | Ethnically diverse children (aged 3-10) from low-income families in urban Uganda and rural Nepal with diverse tribes and migration backgrounds |
| **Impact evidence**  **(*d*=effect size)** | **Efficacy evidence for *ParentCorps*** (2 RCTs)   - Teachers: EBI practices (*d* =.42-.85) - Children: Mental health at post and 3 years post intervention (*d*=.44-.56) - Parents: EBI practices *(d*=.16-.50) | **Efficacy evidence for PD** (immediate post intervention)   - **Uganda** (RCT): Teachers: EBI practice (*d*=.55-1.03); Children: Mental health (*d*=.39-1.08); and Parents: EBI practices (*d*=.22-.56 through knowledge sharing). - **Nepal** (pre-post): Teachers: EBI practices (*d*=.32-.69); Children: Mental health (*d*=.20-.47), school functioning (*d*=.26-.43); Parents: EBI practices (*d*=.43-.79) |

***Note.*** See references in the paper [13, 17, 24, 28, 29, 96, 97]

***Table S2. Power Estimation for Child and Teacher Effectiveness Outcomes***
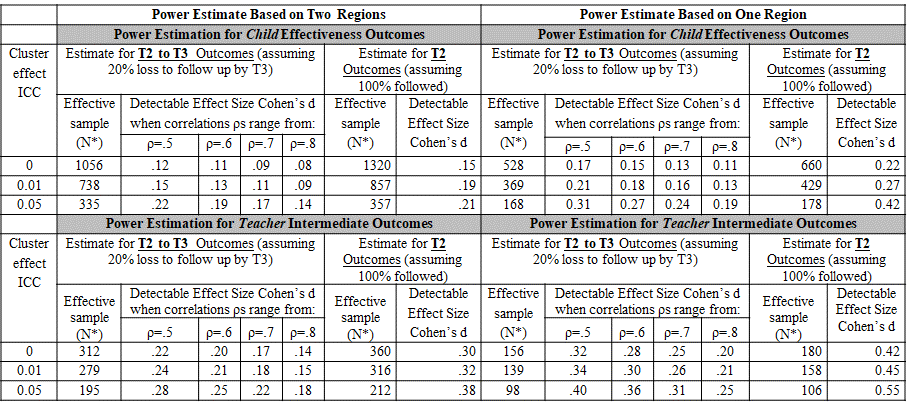

Supplement: Supplementary file 1 — Additional file 1: Table S1. ParentCorps implementation models and impact evidence. Table S2. Power estimation for child and teacher effectiveness outcomes. [file 13033_2022_538_MOESM1_ESM.docx]
